# Digital printing of a novel electrode for stable flexible organic solar cells with a power conversion efficiency of 8.5%

**DOI:** 10.1038/s41598-021-93365-8

**Published:** 2021-07-09

**Authors:** S. Wageh, Mahfoudh Raïssi, Thomas Berthelot, Matthieu Laurent, Didier Rousseau, Abdullah M. Abusorrah, Omar A. Al-Hartomy, Ahmed A. Al-Ghamdi

**Affiliations:** 1 Department of Physics, Faculty of Science, K. A. CARE Energy Research and Innovation Center, King Abdulaziz University, Jeddah, Saudi Arabia; 2 KELENN Technology, 6 rue Ampère, Igny, France; 3 Electrical and Computer Engineering Department, College of Engineering, K. A. CARE Energy Research and Innovation Center, King Abdulaziz University, Jeddah, Saudi Arabia; 4 Physics and Engineering Mathematics Department, Faculty of Electronic Engineering, Menoufia University, Menouf, 32952 Egypt

**Keywords:** Energy harvesting, Renewable energy

## Abstract

Poly(3,4-ethylenedioxythiophene) polystyrene sulfonate (PEDOT:PSS) mixed with single-wall nanotubes (SWNTs) (10:1) and doped with (0.1 M) perchloric acid (HClO_4_) in a solution-processed film, working as an excellent thin transparent conducting film (TCF) in organic solar cells, was investigated. This new electrode structure can be an outstanding substitute for conventional indium tin oxide (ITO) for applications in flexible solar cells due to the potential of attaining high transparency with enhanced conductivity, good flexibility, and good durability via a low-cost process over a large area. In addition, solution-processed vanadium oxide (VOx) doped with a small amount of PEDOT-PSS(PH1000) can be applied as a hole transport layer (HTL) for achieving high efficiency and stability. From these viewpoints, we investigate the benefit of using printed SWNTs-PEDOT-PSS doped with HClO_4_ as a transparent conducting electrode in a flexible organic solar cell. Additionally, we applied a VOx-PEDOT-PSS thin film as a hole transporting layer and a blend of PTB7 (polythieno[3,4-b] thiophene/benzodithiophene): PC71BM (phenyl-C71-butyric acid methyl ester) as an active layer in devices. Zinc oxide (ZnO) nanoparticles were applied as an electron transport layer and Ag was used as the top electrode. The proposed solar cell structure showed an enhancement in short-circuit current, power conversion efficiency, and stability relative to a conventional cell based on ITO. This result suggests a great carrier injection throughout the interfacial layer, high conductivity and transparency, as well as firm adherence for the new electrode.

## Introduction

Organic solar cells (OSCs) are receiving increasing attention and represent an important class of solar technology. Recently, rapid progress has been made towards commercializing organic solar cells owing to their exceptional properties, such as excellent low-light performance, low cost, mechanical flexibility, low weight, free-form design, and semitransparency^1–8^. Furthermore, OSCs are suitable for various applications, including windows, facades, automobile integration, mobile consumer electronics, indoor installations, and smart wearable devices. Today^9–14^, this technology is growing continuously with the development of materials and interface engineering, which has led to enhanced efficiency, with the power conversion efficiency (PCE) for OSCs exceeding 18%^15–17^. One aspect of this research has focused on developing novel transparent conductive electrodes (TCEs) and interfacial layers^18–22^, which is worthwhile due to the potential of such materials to improve OSC efficiencies and stability and add a new road for fabrication. The new transparent conductive electrode (TCE) and modification of the interface layer as a new HTL are essential for balancing the collection of electrons and holes from the active layer by the two types of charge collectors. Additionally, it can be considered a critical property for device structure design^23,24^. Traditional ITO is not appropriate for application in flexible organic devices because under mechanical stress, it is brittle and easily cracks. Many studies have been carried out to find suitable electrode structures for flexible organic solar cells that give performance comparable to that of standard devices. The most famous examples of transparent conductive electrodes that are suitable for flexible organic solar cells are CNTs^25,26^, graphene^27,28^, AgNW^29–36^ and Al-doped ZnO^37^. In addition to these structures, any type of material can be deposited or mixed with metal oxide nanoparticles. Additionally, poly-3,4-ethylene dioxythiophene: polystyrene sulfonate (PEDOT: PSS), which is the cornerstone of this work, is a promising material for a transparent electrode^5,38–40^. PEDOT: PSS is commercially available with different conductivity grades as an aqueous dispersion, and, hence, can be deposited as a thin film via various methods, such as spin coating, dip coating, and printing techniques. In addition, PEDOT:PSS possesses reliable conductivity, a high work function, and high transparency in the visible region. These properties make PEDOT:PSS promising for application as a transporting layer or electrode in solar cells. One of the important features of PEDOT:PSS is that its optical and electrical properties can be modified in various ways using simple chemical or additive methods and posttreatments. From this aspect, various thin films of PEDOT:PSS with different additives have been fabricated, such as CNT + PEDOT and AgNW + PEDOT^34,35,41–43^. These structures have given good performance for flexible PV devices fabricated on PET substrates. The value of the sheet resistance and transmittance and adhesion of PEDOT:PSS to PET for these devices requires more research to improve PV efficiency and stability. In this context, we doped PEDOT-SWNT films with HClO_4_ (0.1 M), which helped to increase the adherence, conductivity (Rs < 10 Ohm/sq), and high transmittance (T > 92% at 550 nm) compared to the reference (ITO electrode) and PEDOT-SWNTs without acid doping. The doping of PEDOT:PSS using inorganic and organic acids such as acetic acid, butyric acid, oxalic acid, hydrochloric acid, propionic acid, and sulfurous acid was proposed in 2010^44^. This doping led to a pronounced improvement in PEDOT:PSS conductivity, which exceeded 200 S cm^-1^. This enhancement of conductivity was attributed to the conformational changes of the PEDOT chains and loss of PSSH from the PSS by acid assistance^45^. Herein, a PEDOT-SWNTs film doped with (0.1 M) of HClO_4_ is applied as an electrode for electron collection on a PET substrate for the first time. For the application of PEDOT-SWNTs + (0.1 M) HClO_4_ as an electrode, intermediate layers were inserted between the electrodes and the active layer to promote carrier extraction/injection and decrease carrier recombination by enhancing the internal electric field and improving energy level alignment at the interface. Specifically, we used zinc oxide (ZnO) as an electron transport layer due to its strong stability and high performance. On the other hand, for hole transportation, we used VOx-PEDOT-PSS as a hole transporting layer. Much research has been done to achieve a satisfactory hole transport layer (HTL) using transition metal oxides. In particular, oxides of molybdenum^46–48^, tungsten^49^, nickel^50,51^, and vanadium^52–55^ have been broadly applied as HTLs in OSCs due to their excellent hole-transporting properties, thermal and chemical stability, large bandgap, and high work function. High vacuum techniques were first applied to grow transition metal oxides, which are expensive and onerous. After that, sol–gel processing^55,56^ was applied to form metal oxides as a good alternative to avoid the complications of vacuum procedures and high thermal treatment; this process enables the formation of thin metal oxide films with low thermal treatment and is suitable for flexible substrates. One of the oxides, specifically VOx, has attracted remarkable attention due to its cost-effectiveness and excellent thermal and chemical stability. Many studies have used VOx with different compositions as a transporting layer^57–59^. Recently, VOx/PEDOT:PSS bilayer HTLs have been used to enhance device performance by smoothing the VOx surface^52–55,57–59^. In this context, Tan et al.^60^ proved the formation of a VOx structure by using XPS measurements for vanadyl acetylacetonate (VO(acac)_2_) spin coated and annealed at 150 °C for 20 min.

To enhance the photovoltaic performance and stability of the solar cells, we used a new processing-based vanadyl acetylacetonate (VO(acac)_2_) ethanol solution doped with a small amount of PEDOT-PSS to form a VOx-PEDOT-PSS film as the HTL. The VOx-PEDOT (3:1 w/w) aqueous solution is a very low-cost process that is not toxic and can be printed with new DMD technology^61–63^ accompanied by thermal annealing at 110 °C for 20 min. In this method, no sol–gel synthesis or post-aging operation is required, which is quite consistent with flexible plastic substrate and printing techniques along with roll-to-roll manufacturing. Furthermore, the VOx-doped PEDOT-PSS (3:1) HTL is highly transparent in the visible region and displays improved hole transport properties.

This work presents a novel arrangement of efficient and stable flexible inverted organic solar cells using the digital materials deposition (DMD) process^33,63^. The bottom electrode, electron transporting layer and VOx-PEDOT film interface layers were all printed with DMD except for the active layer (deposited by spin coating,) and the Ag top electrode (deposited by evaporation). Two devices were fabricated using undoped and HClO_4_ (0.1 M) doped printed PEDOT-SWCNT films on a PET substrate. A third device was fabricated using ITO and used as a reference device. A bulk heterojunction (BHJ) composed of PTB7 and a type of C71-based fullerene derivative material PC_71_BM) was used as an active layer and sandwiched between digital printed ZnO (electron transporting layer) and VOx-PEDOT (hole transporting layer) layers. In addition, 3% of 1,8-diiodooctane (DIO) was utilized as an additive for the active layer owing to its relatively high boiling point to confirm the complete solubility of the BHJ materials. The devices were completed by evaporation of Ag (t = 100 nm) as a cathode. Good photovoltaic performance was obtained with the device based on a PEDOT-SWCNT electrode doped with 0.1 M HClO_4_ with Jsc = 20.06 mA/cm^2^, FF = 0.54, and PCE = 8.60%. PEDOT-SWCNTs doped with (0.1 M) HClO_4_ were found to be an effective replacement for ITO as electrodes with high transmittance, low cost and low sheet resistance. Moreover, a VOx-PEDOT aqueous solution presents a good alternative process for printing a very efficient hole transport layer. This device exhibited a good photoconversion efficiency (PCE = 8.6%) with very strong wettability and flexibility, which was better than that obtained for devices fabricated on ITO electrodes. This process is particularly attractive for fabricating efficient, stable, and flexible organic solar cells. We believe that PEDOT-PSS:SWCNTs + (0.1 M) HClO_4_ solution-processed conducting transparent films and VOx-PEDOT-PSS as an HTL can be successfully incorporated into efficient optoelectronic devices via cost-effective and scalable roll–to–roll or inkjet printing procedures.

## Experimental details

### Materials

Highly purified SWCNTs produced by an arc discharge process and purified by thermal and acid treatments were purchased from NanoIntegris Company and used as received. The diameter and length of the SWNTs were 1.5 ± 0.3 nm and 2 ± 0.5 µm, respectively. PEDOT-PSS (PH1000) aqueous solution was purchased from Heraeus, perchloric acid (HClO_4_) and vanadyl acetylacetonate (VO(acac)_2_) in ethanol solution, PTB7 (poly [[4,8-bis[(2-ethylhexyl)oxy]benzo[1,2-b:4,5-b']dithiophene-2,6-diyl][3-fluoro-2-[(2-ethylhexyl)carbonyl]thieno[3,4-b]thiophenediyl]]]), PC_71_BM (phenyl C71 butyric acid methyl ester) and 1,8 diiodoctane (DIO), chlorobenzene (CB) and dichlorobenzene (DCB) were purchased from Sigma Aldrich. The ZnO nanoparticles solution was obtained from Gene’s ink.

### Technology

The DMD printing technology used in this work is a hybrid digital additive technique that is compatible with a wide range of ink viscosities (ranging from 1 cP to more than 60,000 cP). In addition, DMD technology is suitable for all solvents and reduces material consumption (for example, with 200 µL of solution, we can print more than 50 devices). Thus, DMD technology presents a good alternative as an innovative digital printing technology for all organic devices.

### Device fabrication

**Figure 1:**
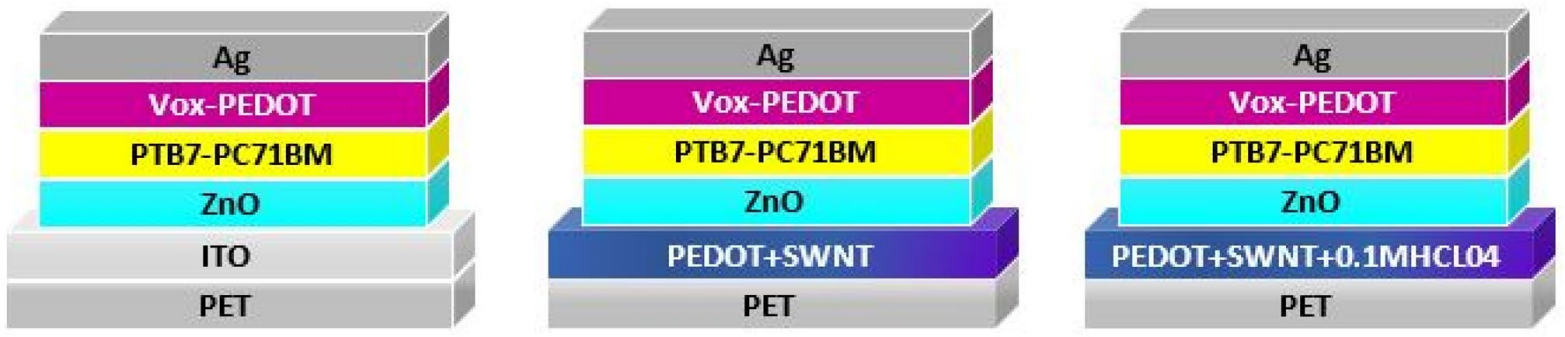
Schematic device structure based on PET/PEDOT-SWCNT, PET/PEDOT-SWCNT + (0.1 M) HClO_4_ and ITO cathodes.

In this work, three architectures for solar cells were constructed on PET substrates, as shown in Fig. 1. The substrates were cleaned with acetone, ethanol, and deionized water in succession with the aid of ultrasonic waves for 15 min followed by a UV-ozone treatment for 20 min. The structures of the three devices were as follows: PET/ITO/ZnO/PTB7-PC71BM/VOx-PEDOT/Ag (references), PET/PEDOT-SWCNTs/ZnO/PTB7-PC71BM/VOx-PEDOT/Ag, and PET/PEDOT-SWCNTs + (0.1 M) HClO_4_/ZnO/PTB7-PC71BM(1:1.5)/VOx-PEDOT/Ag. We started with optimization of the mixing of the SWCNTs with PEDOT-PSS (PH1000) (wt%/wt%). These solutions were ultrasonically treated using a horn-type sonicator (ULS-700S) for 5 min and tip sonication at 30% of the maximum output power (700 W). Following centrifugation for 90 min at 5500 rev/min, the upper 80% of solutions in their containers were collected to obtain well-dispersed SWCNTs in PEDOT-PSS suspensions free of aggregates. UV–Vis–NIR spectroscopic measurements were conducted for the decanted solution, giving its absorbance. The best ratio for the PEDOT/SWCNTs (wt/wt) was obtained (10:1). This solution was printed using a new printing technology named digital materials deposition “DMD” with a printing pressure of 5 kPa, printing speed of 2000 mm/min, step size of 100 µm and flatbed temperature of 40 °C. During the printing process, we used a nozzle diameter of 80 µm, which is suitable for the PEDOT-SWNTs ink viscosity (18 cP). This deposition was carried out without any share force, unlike the inkjet printing process. Consequently, DMD technology produces good films without any coffee rings and with a smoothed surface. The sheet resistance and transmittance of PEDOT-SWCNTs films with increasing thickness (t = 100 nm ± 10 nm) were improved compared with that of ITO/PET (from Rs = 50 Ω/□, T = 88% to Rs = 30 Ω/□, T = 88%). This film was doped with the printing of HClO_4_ (0.1 M) aqueous solution using the DMD technology process and then annealed at 100 °C for 10 min. Then, a ZnO nanoparticle solution was printed at 40 °C with the DMD process and annealed at 120 °C for 10 min (t = 80 nm). After that, the substrates were transferred into a glovebox for coating of the active layer PTB7:PC71 MB (1:1.5 w/w in o-DCB) with 3% DOI in chlorobenzene spin-coated at 1200 rev/min for 60 s (t = 140 nm). For the hole transport layer, we used a mixed solution of vanadyl acetylacetonate (VO (acac)2) with PEDOT-PSS (PH1000) at a weight ratio of 3:1. The VOx-PEDOT film (t = 80 nm) was printed using the DMD process at 40 °C and then annealed at 120 °C for 10 min. Finally, the samples were transferred into a vacuum chamber for Ag (t = 100 nm) deposition. The active area of each device was 0.8 cm^2^.

The sheet resistance (Rs) of the films was characterized using a four-point probe at room temperature, and the optical transmission (T%) and absorption (Abs) were evaluated at a wavelength of 550 nm using a UV–Vis–NIR spectrophotometer (PerkinElmer Lambda 750). Raman spectra were obtained using the 514 nm line of an argon-ion laser as the excitation source at a power of 4.0 µW. Raman spectra were recorded with a resolution of 1 cm^-1^ at room temperature. Scanning electron microscopy (SEM), atomic force microscopy (AFM) and transmission electron microscopy (TEM) investigations were used to evaluate the surface morphology of the new electrode, hole transporting layer and active layer. The current density–voltage (J-V) characteristics for the fabricated flexible organic solar cells under illumination of one sun were measured under AM 1.5G solar irradiation at 100 mW/cm^2^ using a programmable Keithley 2400 source meter. External quantum efficiency (EQE) characterization was achieved using a system combining a monochromator, xenon lamp, lock-in amplifier, and chopper along with a calibrated silicon photodetector.

## Results

**Figure 2:**
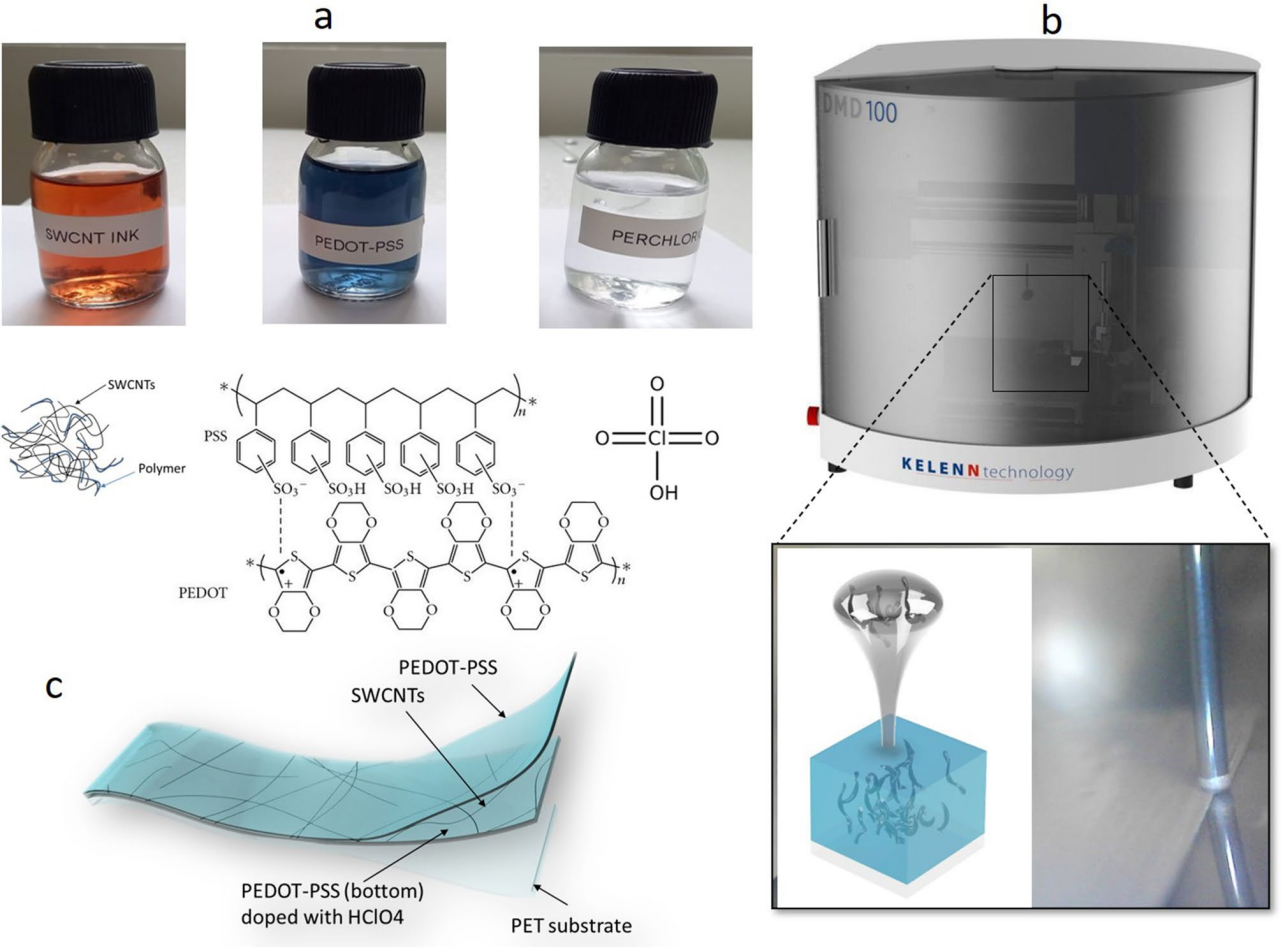
(**a**) Schematic illustration for the materials (SWNTs; PEDOT-PSS; HClO_4_), (**b**) the digital materials deposition (DMD) machine used for the printing process. (**c**) Schematic illustration of PEDOT-SWCNTs (10:1) films doped with (0.1 M) HClO_4_ digital printed on PET substrates.

**Figure 3:**
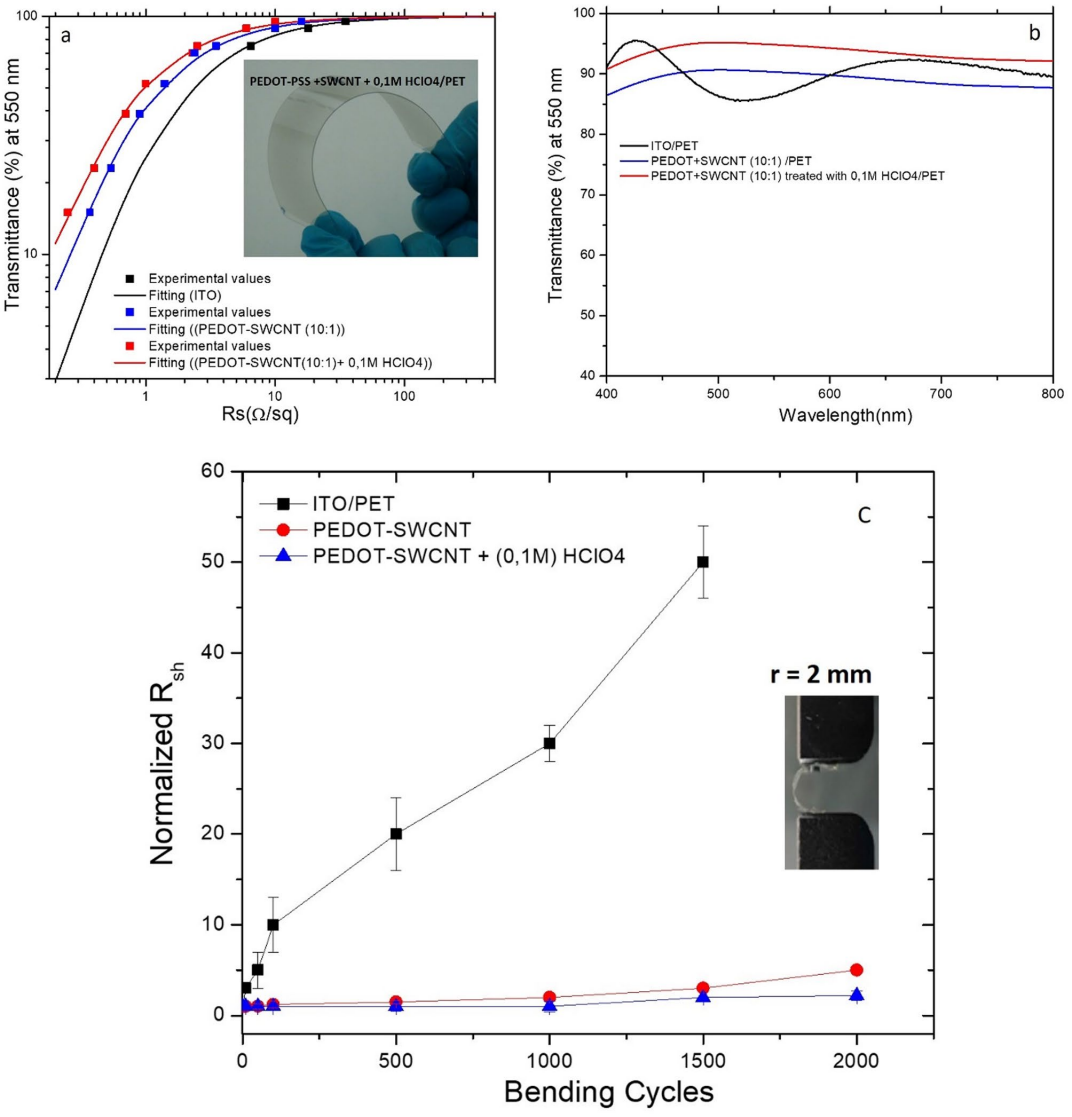
(**a**) Transmission (T at λ = 550 nm) versus sheet resistance (Rs) measurements for ITO/PET (black rectangle) compared to the properties of a PEDOT-SWNTs film on a PET substrate (blue rectangle), PEDOT-SWNTs + (0.1 M) HClO_4_ (red rectangle). The solid line represents a fit to the experimental values obtained for the films on PET. Insert shows optical images of PEDOT-SWNTs + (0.1 M) HClO_4_ on a PET substrate. (**b**) Transmittance curves vs wavelength for the three different films (ITO, PEDOT-SWNTs and PEDOT-SWNTs + (0.1 M) HClO_4_); (**c**) Normalized sheet resistance of ITO/PET, PEDOT-SWNTs/PET, and PEDOT-SWNTs + (0.1 M) HClO_4_/PET at various bending cycles. Bending tests were performed at a curvature radius of 2 mm. All bending tests were performed at a bending frequency of 1 Hz under inner bending conditions with a thin layer of PEDOT-SWNTs. Insert shows digital photographs for a PEDOT-SWNTs + (0.1M) HClO_4_/PET electrode in the flat state and with a bending radius of 2 mm.

Recently, PEDOT: PSS has been considered as a promising conducting polymer for use in elaborate flexible, conductive, and transparent electrodes owing to its low cost, smoothness, good environmental stability, high optical transparency, and low redox potential^63–66^. In this context, many processes have been widely used to enhance the conductivity of PEDOT:PSS, such as polyols, alcohols, surfactants, salts, ionic liquids^67^, and organic solvents. To improve the flexibility and stability of films, Zhang et al.^68^ mixed SWNTs with PEDOT-PSS in DMSO as a solvent, which increased the electronic interaction between PEDOT and SWNTs. This mixture of SWNTs-PEDOT:PSS-DMSO films enhanced the electronic mobility with a sheet resistance of 118 Ω/sq at a transmittance (at λ = 550 nm) of 90.5%. Additionally, using acid treatment^44,64–67^ such as sulfuric acid increases the conductivity and adherence of the film on the surface substrate, but this process is not suitable for PET substrates (a very strong pH can destroy the plastic substrate) and printing processes. Recently, many studies have been carried out to solve this problem, including treatment using novel doping with a small amount of (0.1 M) HClO_4_ diluted in an aqueous solution^45^. This treatment process allowed us to obtain the best electrical and optical properties for these electrodes (30 Ω/sq with a transmittance of 94% at 550 nm). Based on the different techniques mentioned above, this work inspired a new strategy based on a new eco-friendly process, without harmful fog generation and compatibility with inkjet and roll-to-roll printing technology for printing a new transparent conductive electrode with high flexibility wettability and stability. Figure 2 shows the materials and the process used based on mixing SWNTs with an aqueous solution of PEDOT-PSS to form the SWNTs-PEDOT film and treatment by printing of an aqueous solution of HClO_4_ (0.1 M) at room temperature followed by annealing at 110 °C for 10 min. Figure 3 shows the characteristics of the sheet resistance versus optical transmittance (at λ = 550 nm) for PEDOT-PSS: SWNTs (10:1) and PEDOT-PSS: SWNTs + (0.1 M) HClO_4_ films on a PET substrate compared to that for ITO/PET. The best values obtained for the sheet resistance and transmittance of the films were 25 Ω/□ and 8 Ω/□ at 94% and 94% (at λ = 550 nm) (σ_DC_/σ_Op_ ~ 220 and 205), respectively.

### Characteristics of the films

#### Mechanical robustness investigation

We studied the mechanical robustness of the different films, which is substantial for flexible organic solar cells. Figure 3C shows the normalized sheet resistance during the bending cycles of different films. After flexing tests for the films during 2000 cycles with a radius of 5 mm, the bending test results clearly show that ITO is much more vulnerable to mechanical stress than PEDOT-SWNTs and PEDOT-SWNTs doped with (0.1 M) HClO_4_. The sheet resistance of ITO/PET increased after a few bending cycles at a bending radius of 5 mm. However, doped PEDOT-SWNTs displayed noticeably more robust mechanical flexibility than ITO and exhibited stable conductivity with a bending radius below 5 mm. We found that the PEDOT-SWCNTs film doped with (0.1 M) HClO_4_ printed by DMD possesses enhanced mechanical robustness compared with PEDOT-SWNTs and ITO films. This is ascribed to the good intimate contact at the interface realized by forming H bonds between the PET substrate and PEDOT-SWNTs film after HClO_4_ treatment, which illustrates the enhanced mechanical stability and conductivity of PEDOT-SWNTs + (0.1 M) HClO_4_ compared with that of ITO/PET and PEDOT-SWNTs/PET films.

#### Raman spectroscopy

**Figure 4:**
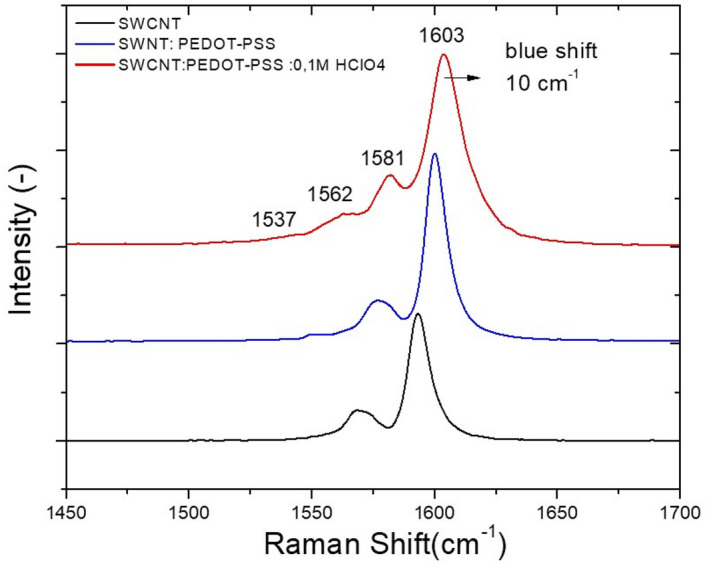
Raman spectra for SWNTs (black line), PEDOT-SWNTs (blue line), SWNTs-PEDOT + (0.1 M) HClO_4_ (red line).

Many works have been carried out to investigate the charge transfer in doped SWNTs by using the G and G0 bands^68–73^. For chemical dopants, charge transfer from the π system of SWNTs into electron acceptor dopants stiffens the bonds and results in a blueshift of the Raman peaks^70–74^. Charge transfer from dopants into the π system of SWNTs softens the C–C bonds, leading to a redshift of the Raman peaks. Figure 4 shows the Raman spectrum analysis of PEDOT-SWNTs + (0.1 M) HClO_4_, PEDOT-SWNTs, and SWNTs.

Several works have pointed out that PEDOT-PSS can dope carbon nanotubes, leading to a variation in the Fermi level within the carbon nanotube that can improve the intertube junction resistance. This resistance improvement can be attributed to the difference in electron withdrawing and donating capability in the doped polymers^75^. Figure 4 reveals two bands at 1571 cm^−1^ and 1592 cm^−1^ that are attributed to G0 and G-bands of the SWNTs, respectively. It can be noted that the G-bands are slightly upshifted (blueshift) by approximately 5 cm^−1^ for the PEDOT-SWNT sample relative to the bare nanotube. This upshift can be attributed to the phonon stiffening effect due to doping^75^. The wrapping of PEDOT-PSS around the nanotubes can improve the load charge transfer from PEDOT to SWNTs, and, consequently, improve the sheet resistance (30 Ω/sq) compared to the PEDOT-PSS film alone (100 Ω/sq) for the same transparency (T = 88% at 550 nm). However, from the spectra for PEDOT-PSS:SWNTs, we detected the existence of another peak at nearly 1555.6 cm^−1^. This peak was attributed to the presence of another interaction between SWNTs and the (–SO3H) group of PSS. Treatment with HClO_4_ (0.1 M) for doping the PEDOT-SWNTs film (Fig. 4) leads to a blue further blueshift of the G0 and G-bands at 10 cm^−1^, located at 1581 cm^−1^ and 1603 cm^−1^, respectively, relative to SWNTs, and shifts of approximately 5 cm^−1^ relative to their positions before treatment. Additionally, the effect of doping leads to the appearance of a new peak at 1537 cm^-1^. This new peak can be attributed to the presence of chlorine oxygen bonds in the structure due to treatment with the HClO_4_ inorganic acid, which possesses ultrahigh acidity (pKa ≈ − 10). These bonds lead to a good combination of HClO_4_ molecules with the PSS components of PEDOT: PSS (PH1000) and SWNTs. Therefore, the treatment of the PEDOT-PSS:SWNTs film with a small amount of HClO_4_ aqueous solution improves the PEDOT crystallinity^45^, which improves the conductivity of the films.

#### UV–Vis-NIR spectra

**Figure 5:**
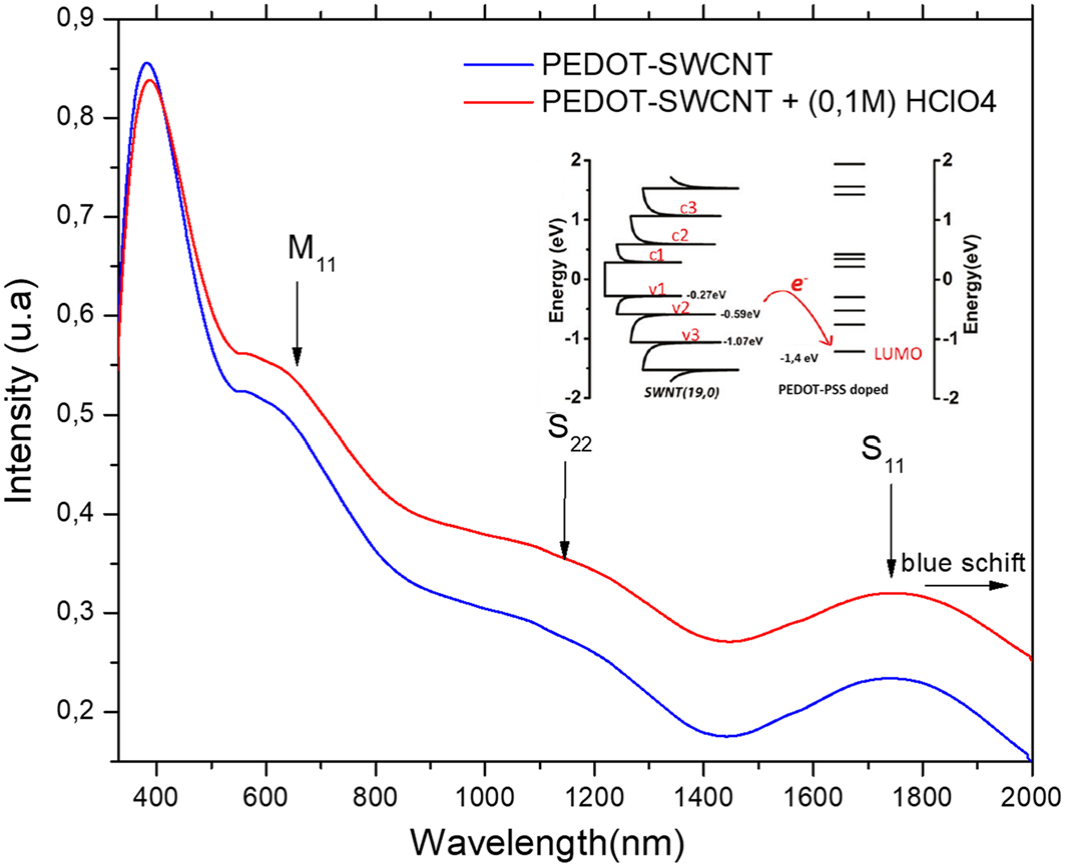
Optical absorption spectra for PEDOT-SWNTs and PEDOT-SWNTs doped with HClO_4_ showing the p-doping effect of PEDOT-SWNTs due to the use of (0.1 M) HClO_4_, which leads to a small blueshift, and the inserted schema illustrate the electron transfer from the SWNTs to PEDOT-PSS.

Figure 5 shows UV–vis-NIR absorption spectra for PEDOT-SWCNTs with and without HClO_4_ treatment printed with DMD technology. For UV–vis-NIR spectroscopic investigation, films were printed on PET flexible substrates. The spectrum shows the S11 (1.8 µm) and S22 (1 µm) peaks, which are ascribed to the interband transitions in the semiconducting tubes between the first and second pairs of van Hove singularities, respectively. Additionally, the spectrum shows the presence of a third M11 (0.7 µm) peak, which is attributed to the intraband transition in metallic CNTs. This result indicates the metallic nature of SWCNTs. Furthermore, the presence of the M11 peak indicates that the electronic structure of SWNTs is not affected by the polymer. Tracking the position of the van Hove nanotube bands in the visible and NIR regions reveals interesting information about PEDOT-PSS and SWCNTs interactions^76^. We found that the S11 and S22 peaks for PEDOT-SWNTs + (0.1 M) HClO_4_ were shifted relative to the positions of these bands in PEDOT-SWCNTs. This result indicates the presence of charge transfer from PEDOT-PSS (0.1 M) HClO_4_ to SWNTs due to the formation of hydrogen and chlorine oxygen bonds and confirms the result obtained from Raman analysis. Therefore, Fig. 5 shows the absorption peak near 400 nm, which is attributed to both π-π* and n-π* transitions in the phenyl ring bond of PEDOT-PSS. In addition, the absorption spectra show quite similar light absorption peaks for PEDT-PSS and SWCNTs despite the ratio of 10:1 wt/wt. Because this result concerns the solution ratio for PEDOT-PSS and SWCNTs, Fig. 5 shows the absorption spectrum of the PEDOT-SWCNTs film after thermal annealing at 130 °C for 10 min. For this reason, the ratio (10:1) should be changed in the film. The concentration of the SWCNTs solution was 0.1 mg/ml.

#### AFM & SEM images

**Figure 6:**
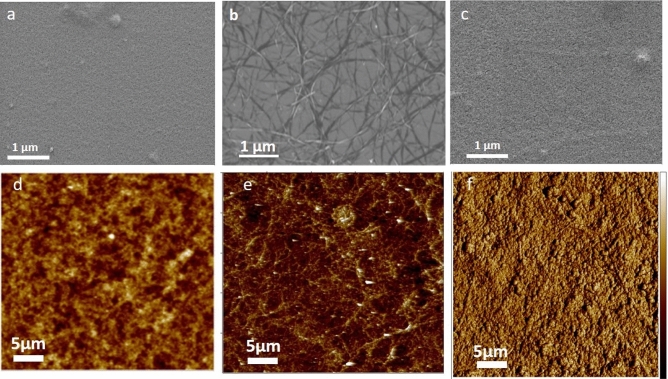
SEM and AFM images of (**a**,**d**) PEDOT-PSS; (**b**,**e**) PEDOT-SWNTs (10:1); (**c**,**f**) ZnO/PEDOT-SWNTs + (0.1 M) HClO_4_.

The morphology of different films composed of PEDOT-PSS, PEDOT-SWNTs, and PEDOT-SWNTs doped with HClO_4_/ZnO was characterized by scanning microscopy (Fig. 6a–c) and tapping mode AFM (Fig. 6d–e). The PEDOT-PSS film's surface roughness (RMS ~ 0.89 nm with a thickness of 70 nm) is very smooth compared to that of doped PEDOT-SWNTs (RMS ~ 10 nm with a thickness of 100 nm). Figure 6b,e show the presence of the nanotube, which reveals the high roughness and film thickness. Therefore, the sheet resistance and transparency of this film were improved by adding a small amount of SWNTs and p-doping with (0.1 M) HClO_4_, which enhanced the crystallinity quality of the film (Fig. 6e). We note the absence of large or small nanotube bundles that is due to fact that the PEDOT-PSS solution mixed very well with the SWNTs aqueous solution and presented a good procedure to form very good films with an ecofriendly process. One of the advantages of DMD technology is that it can be used to print a smooth surface. This deposition is carried out without any shear force, unlike the inkjet printing process. Thanks to this process, we obtained a good film without any coffee rings and with a smooth surface.

The highly conductive and smooth surface of the PEDOT-SWNTs doped with HClO_4_ is the best candidate to replace ITO. Then, the addition of a ZnO nanoparticle film (t = 40 nm) as the electron transport layer introduced a smooth surface with very high transparency.

**Figure 7:**
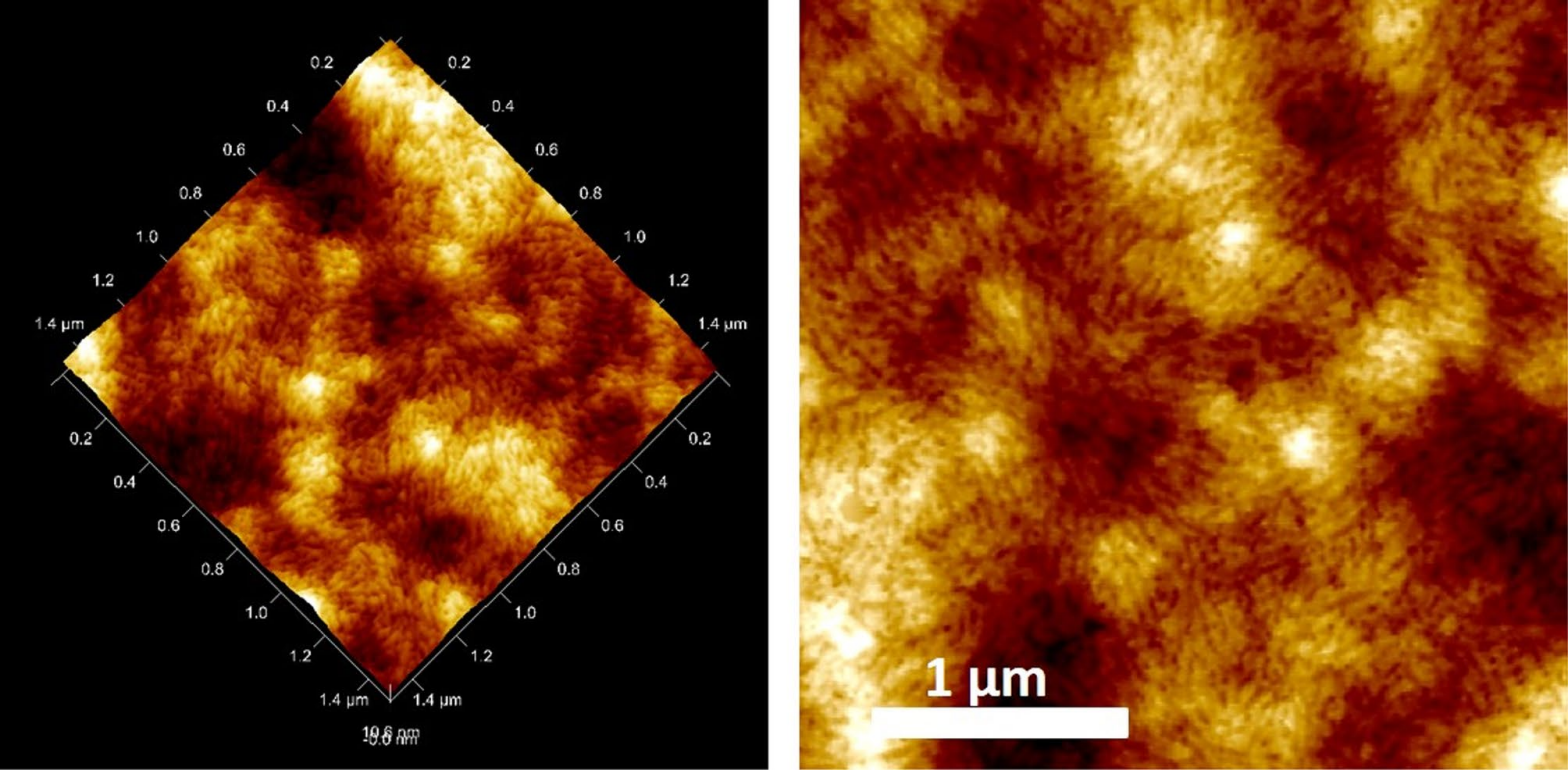
AFM image of PTB7- PC71BM (1:1.5 w/w) under 3D (right) and 2D images (left).

Figure 6c,f show the morphology and roughness of the ZnO/PEDOT-SWNTs-doped film (RMS ~ 4 nm, thickness ~ 160 nm) sheet resistance Rs ~ 12 Ω/sq with T = 94% at 550 nm. The active layer composed of the bulk heterojunction (BHJ) based on PTB7-PC71BM (1:1.5 wt/wt weight) was deposited onto this film in a glovebox. Figure 7 shows the morphology of PTB7-PC71BM with low roughness (RMS = 4 nm) without any PC71BM aggregates and high crystallinity, allowing good photovoltaic performance. The hole transport layer (VOx doped with a small amount of PEDOT-PSS) was printed with DMD technology and annealed at 110 °C, 10 min.

**Figure 8:**
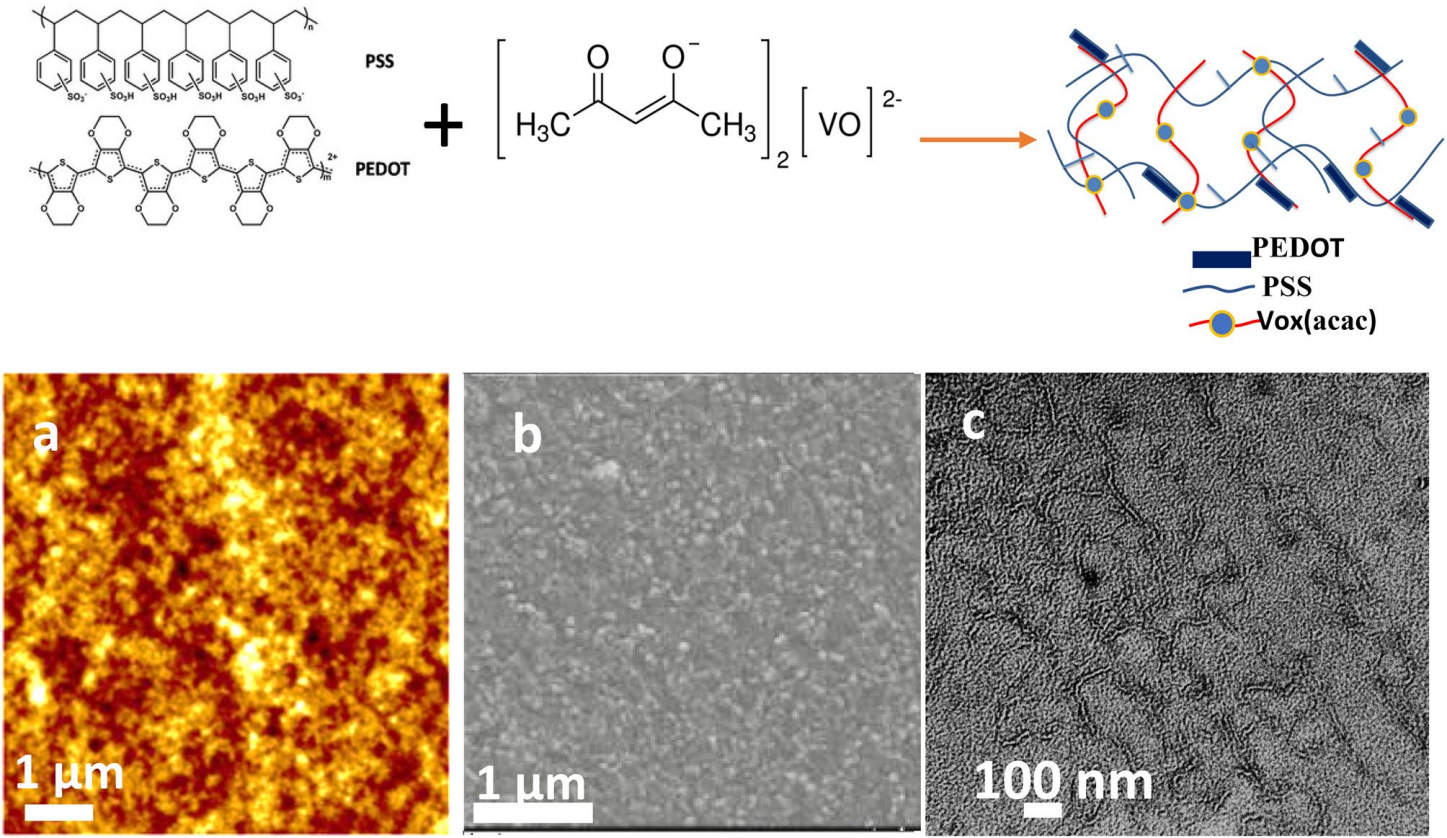
Schematic illustration of the formation of the interpenetrating networks by mixing PEDOT-PSS with VOx(acac). AFM (**a**), SEM (**b**) and TEM (**c**) images of the PEDOT-PSS:VOx (1:3) printed film.

Figure 8 shows the AFM, SEM, and TEM images of a thin layer (t = 80 nm) of PEDOT-VOx; the TEM image shows the PEDOT-PSS chains in the VOx matrix with the occurrence of standing lamellar and nanofibrillar morphologies. The presence of covalent bonds between PSS and vanadyl acetylacetonate has a pronounced effect on the morphology of the film in the lateral direction, limiting phase separation and forming a structure with dimensions of the order of 25 nm. This enables one to improve the mobility of the charges.

### Characterization of the devices

#### Performance of the devices

**Figure 9:**
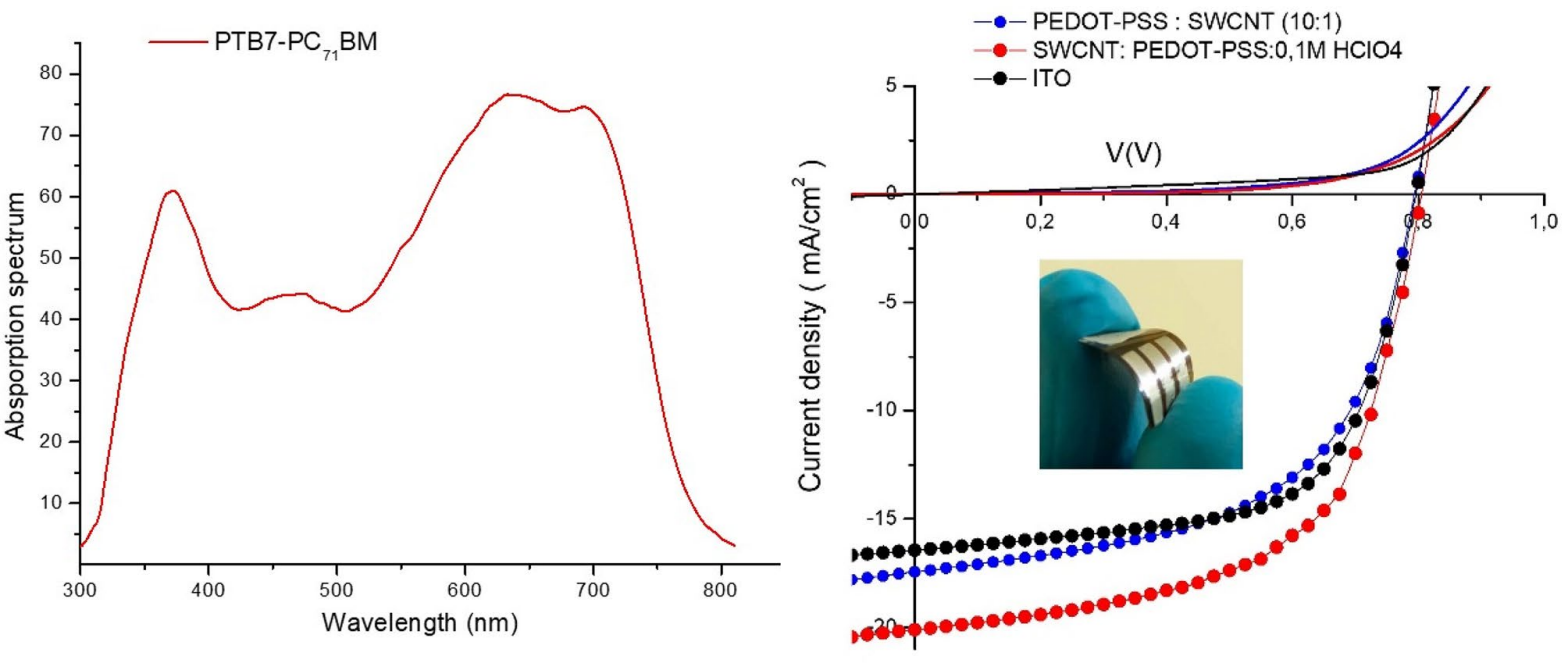
Absorption spectrum of PTB7- PC_71_BM and the J-V curves measured in the dark and under illumination for ITO/ZnO/PTB7-[70]PCBM/VOx-PEDOT/Ag, SWCNTs-PEDOT/ZnO/PTB7- PC_71_BM/VOx-PEDOT/Ag, SWCNTs + PEDOT-PSS + 0.1 M HClO_4_/ZnO/PTB7- PC_71_BM/VOx-PEDOT/Ag.

To explore the effects of PEDOT-SWCNTs doped with HClO_4_ and VOx-PEDOT on device performance, we fabricated three different architectures for flexible organic solar cells: reference solar cell with a structure of PET/ITO/ZnO/PTB7-PC71BM/VOx-PEDOT-PSS/Ag, along with two devices using SWCNTs + PEDOT: PSS(1:10) and SWCNTs + PEDOT-PSS (1:10) doped with (0.1 M) HClO_4_ as a replacement for the ITO film. The three devices included the same active layer, and VOx-PEDOT:PSS (3:1) was used as the HTL and a Ag film was used as the anode. The fabricated cells were characterized under air mass 1.5 global (AM 1.5G) conditions with an irradiation intensity of 100 mW cm^-2^. The characteristic parameters for the fabricated cells are listed in Table 1. As revealed in Fig. 9 and Table 1, the PTB7:PC71BM-based cells with PEDOT-PSS:SWNTs (10:1) as the TCF and VOx-PEDOT:PSS (3:1) as the HTL displayed a PCE of 7.7%. The short-circuit current density (Jsc), open-circuit voltage (Voc), and fill factor (FF) were determined to be 17.45 mA cm^−2^ and 0.79 V, and 56%, respectively.

**Table 1:** Summary of the photovoltaic performances of all devices with the PET/TCF/PTB7:PC71BM/VOx-PEDOT-PSS/Ag structure and an area of 0.8 cm^2^.

TCF	T(%) at 550 nm	σ_DC_/σ_Op_	Voc (V)	Jsc measured (mA/cm^2^)	Jsc calcul ( mA/cm2)	FF (%)	PCE (%)	Rs (Ω.cm^2^)	Rsh (Ω.cm^2^)
ITO	88 (± 2)	202(± 5)	0.79 (± 0.01))	16.25 (± 0.1)	15.95 (± 0.1)	58 (± 0.5)	7.45 (± 0.1)	9.65	8919
PEDOT-SWCNTs (10:1)	90 (± 2)	205 (± 5)	0.79 (± 0.01)	17.45 (± 0.05)	16.85 (± 0.05)	56 (± 0.5)	7.7 (± 0.1)	8.72	5140
PEDOT-SWCNTs (10:1) + (0.1 M)HClO4	92 (± 2)	220 (± 7)	0.80 (± 0.01)	20.06 (± 0.01)	18.86 (± 0.01)	54 (± 0.2)	8.6 (± 0.0)	5.52	8642

**Figure 10:**
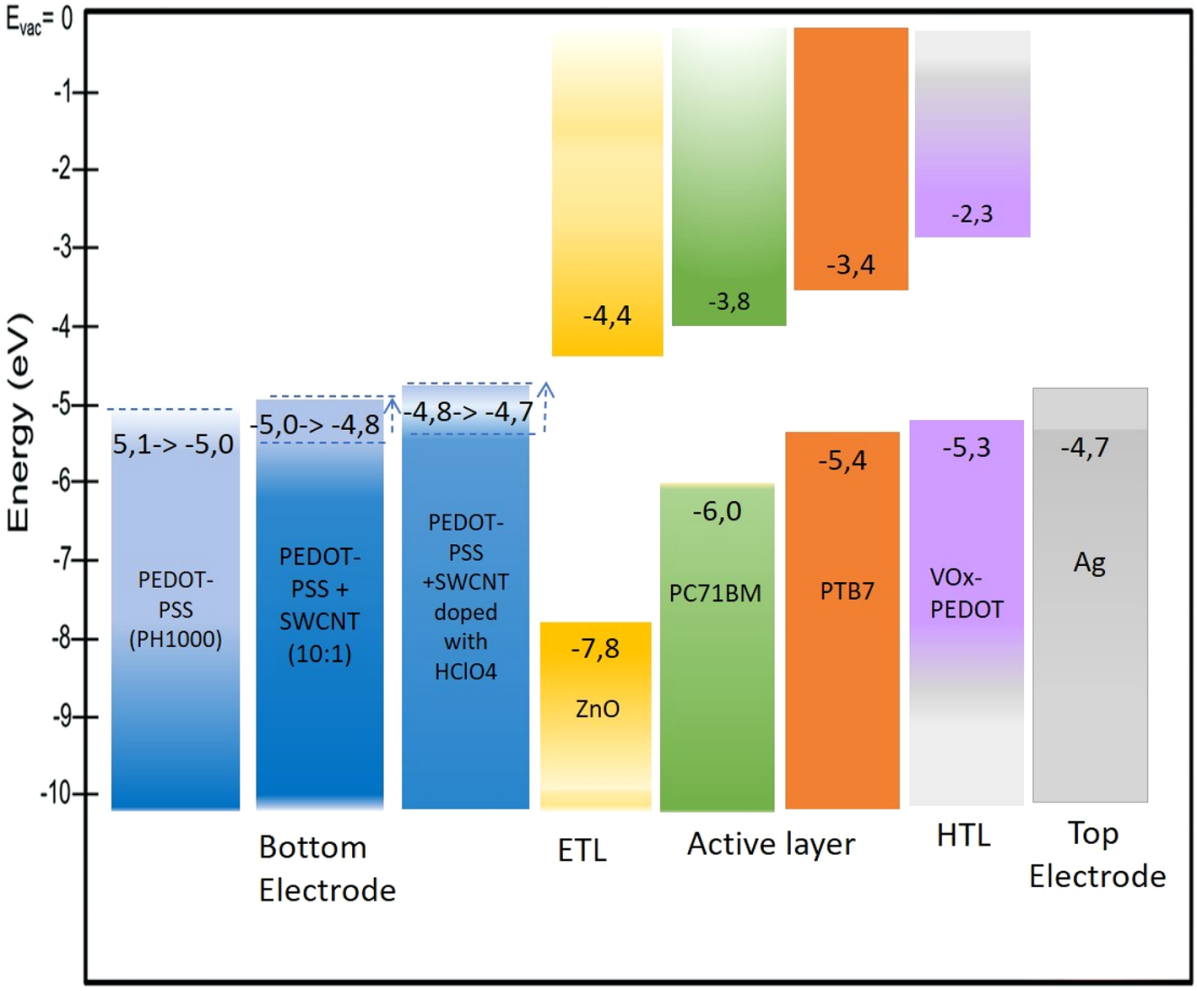
Energy level diagram for PSCs and electrical contacts for the TCF(PEDOT-PSS(PH1000), PEDOT-PSS + SWCNTs and PEDOT-PSS + SWCNTs + HCLO_4_), HTL and HOMO–LUMO of the donor polymers (PTB7) and PC_71_BM.

**Figure 11:**
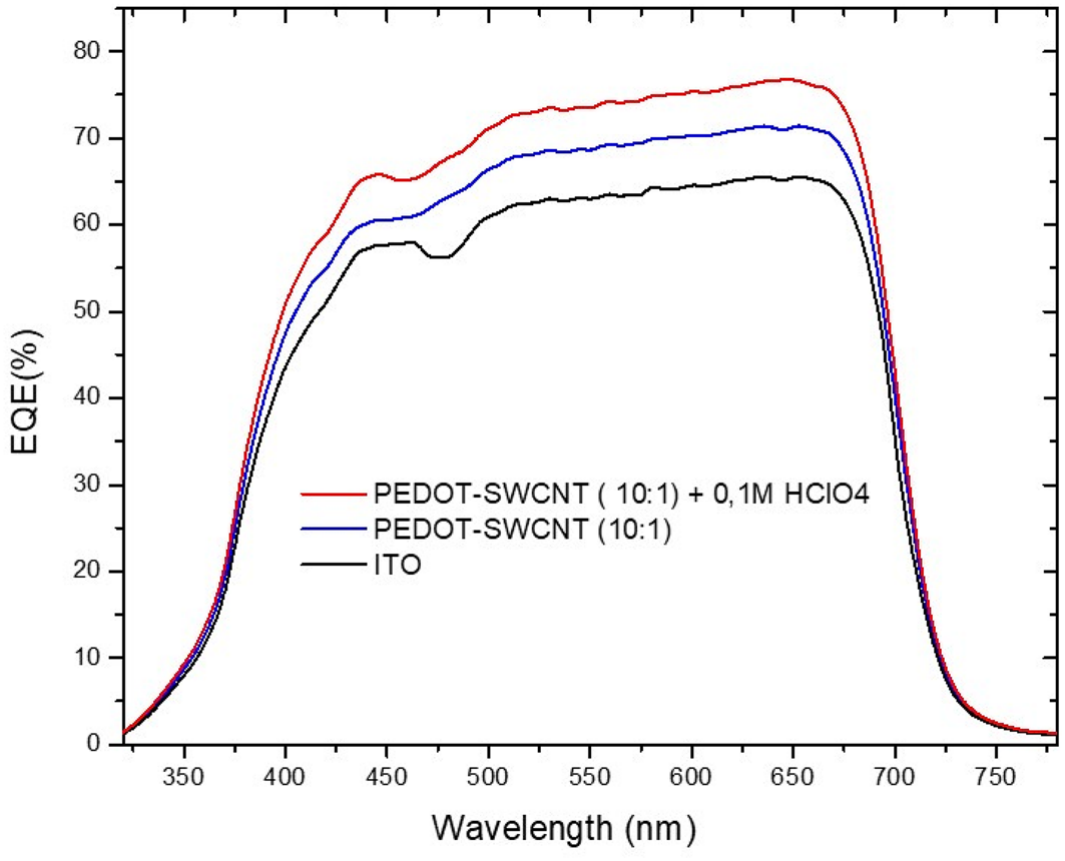
EQE spectra for the three devices based on different types of ITO cathodes, blends of PEDOT- SWNTs and PEDOT- SWNTs + (0.1 M) HClO_4_.

When PEDOT-PSS:SWNTs (10:1) were doped with (0.1 M) HClO_4_ for use as an electron collector, the Jsc, Voc, and PCE concurrently increased to 20.06 mA cm^−2^, 0.80 V and 8.5%, respectively. While the FF slightly decreased to 54%. The cell based on PEDOT-PSS: SWNTs and PEDOT-PSS: SWNTs (10:1) + (0.1 M) HClO_4_ showed an enhanced efficiency relative to a conventional flexible device with ITO as the TCE with PEC = 7.45%. The optimal, efficient device is based on PEDOT-PSS: SWNTs (10:1) + (0.1 M) HClO_4_. These results were confirmed by decreasing the series resistance (Rs) from 8.40 Ω.cm^2^ to 5.50 Ω.cm^2^ and increasing the shunt resistance (Rsh) from 540 Ω.cm^2^ to 8642 Ω.cm^2^ along with improving the transmission of the electrode layer in the visible range. We attributed the enhancement in device efficiency to improved charge injection/extraction at the new transparent conducting film doped with HClO_4_ and minimization of charge recombination at the interface between the HTL and active layer due to a reduced energy barrier, as shown in Fig. 10. The EQE spectrum shown in Fig. 11 for the three devices confirms the superiority of the current density of the device based on the new PEDOT-PSS: SWCNTs (10:1) + (0.1 M) HClO_4_ electrode. This is evident more in the log–log figure for the photocurrent versus effective applied voltage (Veff = V0—V curves). The good efficiency is confirmed by the very low current density measured in the dark for devices printed on the new electrode based on PEDOT-SWNTs doped with HClO_4_ compared to the ITO device.

#### Stability studies

**Figure 12:**
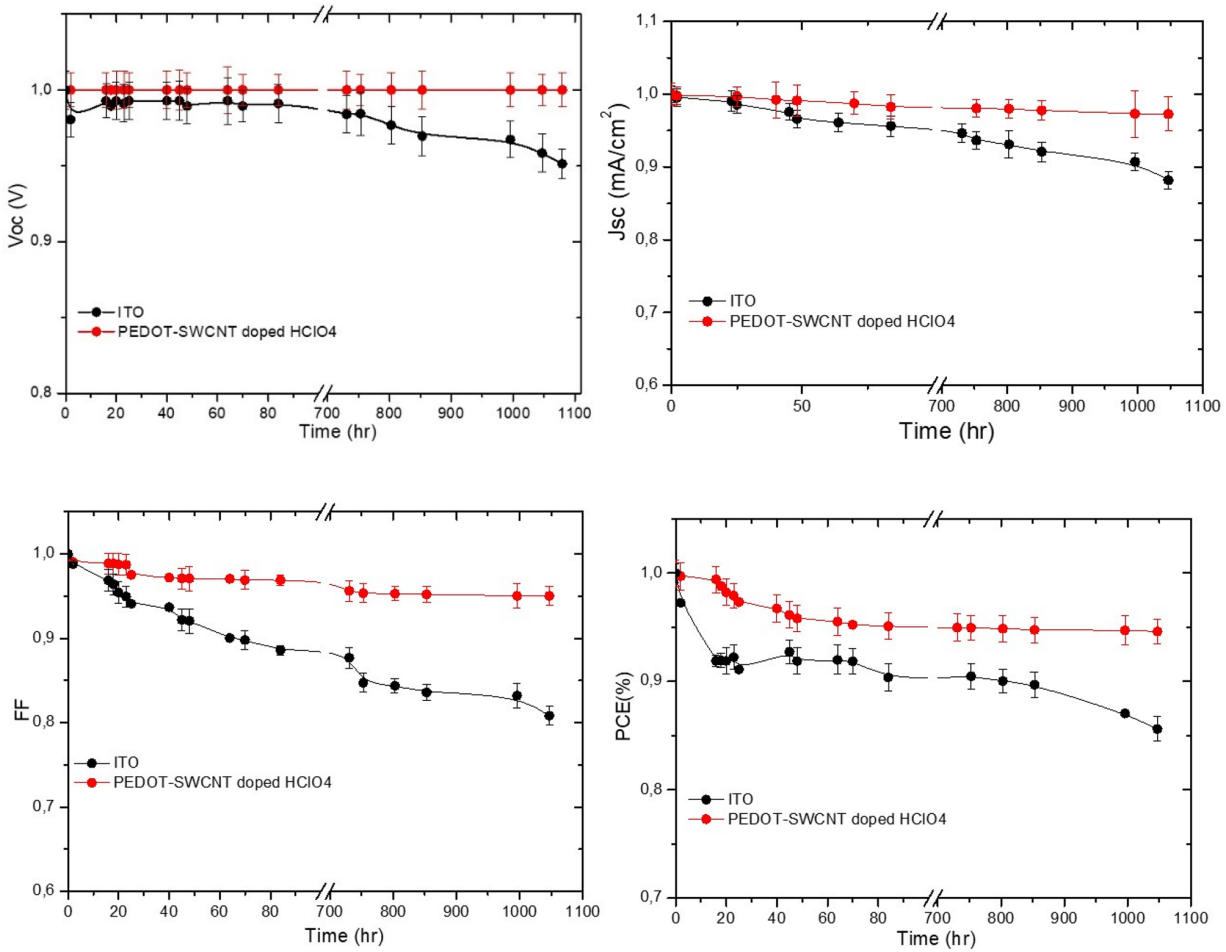
Decay curves under illumination for the Voc, FF, Jsc and PCE (%) for PTB7- PC71BM on ITO/PET (black line) and SWCNTs-PEDOT doped HClO_4_/PET (red line) devices in a glovebox.

The bottom electrode layers of the inverted organic solar cell are among the parts of the device that are highly prone to degradation and have a direct effect on the degradation of the active layer, and consequently, the power conversion efficiency. However, the stability and morphology of the bottom electrodes of the solar cell are the key important active layers for achieving large gains in terms of stability to realize device operation that can be measured in years rather than minutes. In addition, the interface layers of the solar cell based on ZnO and VOx-PEDOT are vulnerable to molecular oxygen and water. The famous mechanisms for photooxidative degradation that can take place in this system are the photooxidation of the organic material that will degrade the electron/hole transport properties, and thus, the photovoltaic performance^77^. The second mechanism is that the structure of VOx-PEDOT/Ag with a low work function can lead to the formation of metal oxides in the interface that will consequently act as a transport barrier, resulting in deterioration of the photovoltaic performance. Many works have studied the photooxidation of flexible organic solar cells in specific device locations by employing time-of-flight secondary ion mass spectrometry (TOF–SIMS) in combination with isotopically labeled atmospheres^78^. Due to sensitivity towards molecular oxygen and water at the interface for PEDOT-SWCNTs doped with HClO4 and VOx-PEDOT of nonencapsulated inverted devices with an architecture of ITO/SWCNTs-PEDOT + 0.1 M HClO4/PTB7-PCBM/VOx-PEDOT/Ag, we studied the stability of our devices in air. We will focus the discussions on the stability of two devices under illumination. After plotting all the curves for the devices, as shown in Fig. 12, there several interesting trends can be observed from the data. The investigation of the stability of devices based on SWCNT-PEDOT doped with HClO_4_ under 1 sun (AM 1.5), 50 °C) revealed a strong stability. This improvement was obtained by employing PEDOT-SWCNTs-doped HClO_4_ in the bottom electrode and the use of VOx doped with a small amount of PEDOT as the HTL and ZnO as the ETL, which allowed an increase in the charge transfer from the active layer to the electrodes and formed a very strong interface towards molecular oxygen and water.

**Figure 13:**
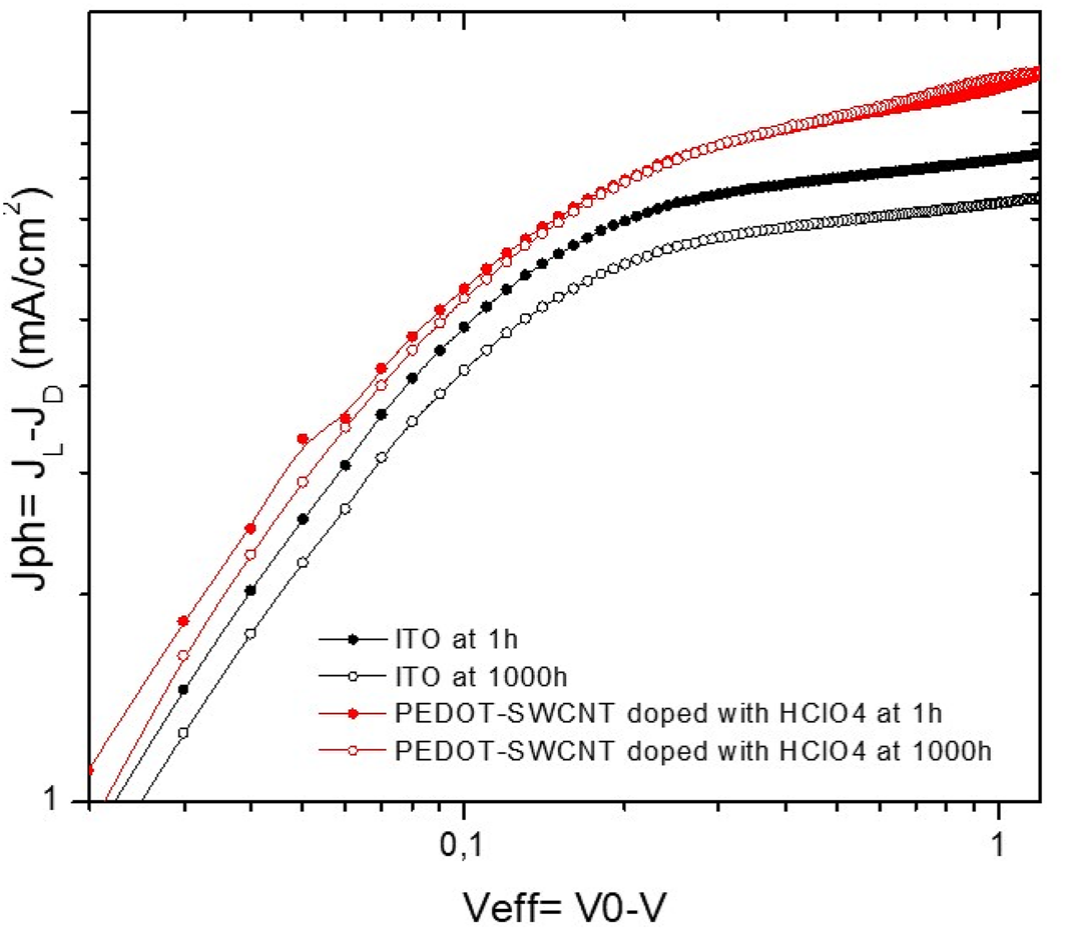
Experimental photocurrents after 1 h and 1000 h as a function of Veff for flexible organic solar cells based on PTB7-PC_71_BM on ITO as a reference (black curves, circular symbol) and on PEDOT-PSS: SWCNTs doped with HClO_4_ (red curves, circular symbol).

Figure 13 shows the measured photocurrent Jph versus the effective applied voltage (Veff = V0–V) for flexible organic solar cells based on ITO and PEDOT-SWNTs doped with (0.1 M) HClO_4_ as electrodes after lifetimes of 1 h and 1000 h. The measured current under illumination (JL) corrected for the dark current (JD) is the photocurrent (Jph = JL -JD). However, the voltage at which the photocurrent Jph is 0 is the compensation voltage V0. The photocurrent is obviously increased linearly with voltage in the range of voltages close to the compensation voltage (V0–V < 0.1 V). The photocurrent enters a regime of a square root dependence on the effective voltage in the range V0–V > 0.1 V. Similar behavior has been observed for BHJ solar cells based on multiple conjugated polymer fullerenes, where the photocurrent decreased for V0–V > 0.1 V and was ascribed to recombination effects^79^. Definitely, the low mobilities or short lifetimes of free carriers (due to either recombination or trapping) restrict the observed photocurrent in flexible organic solar cells.

As evidenced in Fig. 13, after illumination for 1 h, devices printed on PEDOT-SWNTs doped with HClO_4_ on a PET substrate (red curves up circular symbol) constantly yield higher photocurrents in the square root regime (V0–V > 0.1 V) compared to the reference device on ITO (black curves, circle symbol). This result suggests that the new electrode has high conductivity and shows improved charge injection, which leads to a decrease in interfacial recombination. This can be attributed to the good work function of PEDOT-SWNTs + (0.1 M) HClO_4_ and the good new hole transport layer VOx-PEDOT (3:1) with high conductivity and transparency combined with the best electron transport layer of ZnO. All these factors lead to an increase in the interfacial contact between the active layer and the electrodes and improved hole and electron transport. After 1000 h under illumination in a glovebox, Fig. 13 shows a reduced photocurrent for the reference cell based on ITO. On the other hand, after 1000 h, the flexible organic solar cell with a PEDOT-SWNTs + (0.1 M) HClO_4_ electrode shows higher stability than the reference cell. This is due to the good stability of the new electrode with high wettability on the PET substrate and also due to the good interface layer, which allows an increase in the charge injection. In conclusion, this study showed that PEDOT-SWNTs doped with HClO_4_ as a new electrode and VOx-PEDOT as a hole transport layer are a good approach for obtaining a printing flexible solar cell with increased performance and stability.

## Discussion

A flexible bulk heterojunction organic solar cell with a PCE reaching 8.5% was fabricated on a thermoplastic polymer, PET. For optimization of efficiency, stability, and flexibility, three configurations were investigated. The main difference between the three architectures is the type of cathode. Three types of cathodes were applied: ITO, a blend of PEDOT- SWNTs and PEDOT- SWNT + (0.1 M) HClO_4_. ZnO was used as a buffer layer for the electron-transporting and hole-blocking layers for the three devices. On the other hand, PEDOT doped with vanadyl acetylacetonate acts as a hole transporting layer and a high work function silver film is used as an anode. A buffer layer composed of an interfacial hole transporting layer of VOx-PEDOT was used to prevent recombination at the interface between the electrode and active layer and enhance hole selectivity. The rule of using vanadyl acetylacetonate rather than V_2_O_5_ enables one to avoid the complicated process of vacuum deposition and formation of small islands instead of uniform film for a low cost and easy deposition process. The bulk heterojunction active layer consists of a low bandgap polymer that works as a donor, explicitly PTB7, and an acceptor, PC71BM, with a 3% DIO as an additive. The role of DIO addition is to control the aggregation of the PC71BM acceptor in the active layer. In addition, a previous investigation showed that mixing 3% DIO with solvent improved the performance of a solar cell^80^. The absorption of a blend of PTB7/PC71BM possesses strong absorption in the whole visible range. As our cell's active layer is composed of a blend of PTB7/PC71BM, light absorption leads to the formation of a bound electron and hole (Exciton) in PTB7. As the difference between the LUMO of PTB7 and the LUMO of PC71BM is approximately 0.4 eV, this energy is enough to dissociate the exciton at the interface. At this time, are transported through the donor to the hole transporting layer of VOx-PEDOT, and, finally, injected into the Ag anode. This injection is supported by Ag's smaller work function energy to the HOMO level of the VOx-PEDOT intermediate layer, as shown in Fig. 10. On the other hand, electrons can be transferred to acceptor/PC71BM and then to ZnO, where there is no barrier for transportation, as shown in Fig. 10. Finally, electrons are collected by the cathode. In our three arrangements, the three applied cathodes have work functions larger than ZnO; consequently, there is no barrier for electron transport.

The reported performance parameters vary with the three different cathodes applied in this work. Using ITO as the cathode showed the lowest short-circuit current (Jsc), open-circuit voltage (Voc), and power conversion efficiency relative to the other two devices with doped and undoped PEDOT- SWNTs. For the same device based on ITO, the sheet and shunt resistances and fill factor are larger relative to the other devices. Replacing ITO with PEDOT- SWNTs does not affect Voc but results in an increase in Jsc and PCE by 7.4% and 3.4%, respectively. FF is decreased by 3.5%, which is attributed to the roughness of the PEDOT-SWCNT surface comprising the ITO despite the decrease in the series and sheet resistances by 9.2% and 42.3%, respectively. The increase in the performance of the cell based on PEDOT- SWNTs anodes was attributed to the synergistic effect of the polymer and single-wall carbon nanotubes. PEDOT fills the micropores of the SWNTs and forms a continuous contact. In addition, the nanotubes have high electrical conductivity in addition to a large surface area that improves the interfacial contact between the nanotube and polymer. These factors can lead to an enhancement of carrier transportation, as confirmed by the decrease in sheet resistance.

Using PEDOT- SWNTs + (0.1 M) HClO_4_ as a cathode, the performance and stability of the cell was improved relative to the devices based on ITO and PEDOT-SWNTs (shown in Fig. 12). The Voc, Jsc, and PCE increased by 1.3%, 26.8%, and 15.4% relative to the cell based on ITO, while FF, Rs and Rsh decreased by 6.9%, 42.7%, and 3.1%, respectively. The addition of HClO_4_ at a low percentage improved the stability and conversion efficiency of the cell. These improved parameters gear up the utilization of PEDOT- SWNTs + (0.1 M) HClO_4_ as a bottom electrode for efficient inverted organic solar cells. For this cell, the SWNTs improved the conductivity, and the addition of HClO_4_ improved the interfacial contact with the electron transporting layer and enhanced the adhesion of the anode to the PET substrate. Additionally, by comparing the sheet resistance for the cell based on PEDOT- SWNTs and PEDOT- SWNTs doped with HClO_4_, the sheet resistance greatly decreased, reflecting a decrease in the bulk resistance of the bottom electrode. All these factors led to a pronounced decrease in sheet resistance and, consequently, the efficiency of the cell.

**Figure 14:**
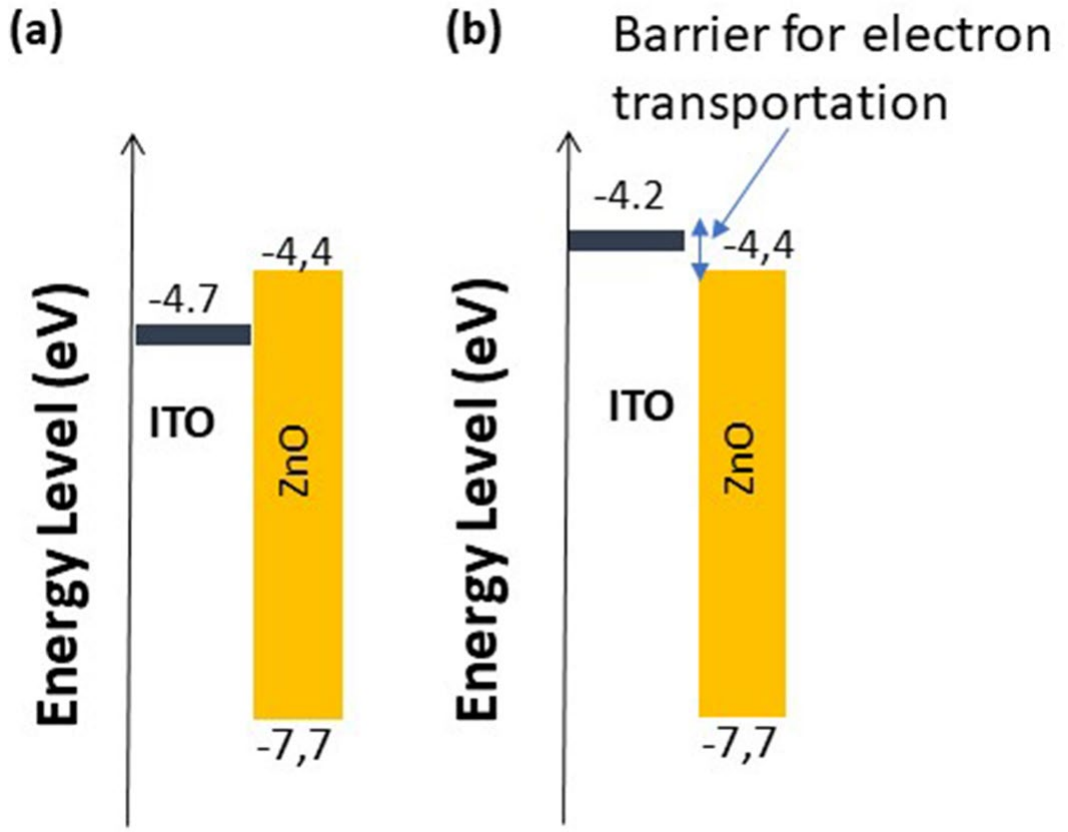
Effect of illumination on the position of Fermi level of ITO.

Regarding the stability under illumination, the proposed solar cell based on the bottom electrode of PEDOT- SWNTs + (0.1 M) HClO_4_ showed better stability relative to the cell based on an ITO cathode. Deterioration of the solar cell based on ITO can be ascribed to a shift of the work function of ITO to a lower energy reaching 4.1 eV. The position of the Fermi level shift to a lower energy results in a change in the junction between ITO and ZnO from a Schottky type to an ohmic type with a barrier to the ZnO electron transporting layer of approximately 0.2 eV, as shown in Fig. 14. A previous investigation showed that continuous UV light illumination or AM 1.5 conditions can lead to a change in the work function of ITO from 4.7 eV to approximately 4.2 eV^81^.

We can conclude that the inverted flexible cell based on PEDOT- SWNTs + (0.1 M) HClO_4_ fabricated with DMD technology is a promising architecture for future commercialization.

## Conclusion

In this study, we successfully printed a highly flexible, transparent electrode based on PEDOT-PSS:SWCNTs (10:1) for direct integration in an organic solar cell using Ag as the anode and ZnO and VOx-PEDOT (3:1) as interface layers (ETL and HTL). Doping PEDOT-PSS: SWCNTs with HClO_4_ allowed us to obtain a very conductive electrode and improved the adhesion with the PET substrate, resulting in a low sheet resistance of 8 Ohm/sq and a high optical transmittance exceeding 92%. By using PEDOT-SWNTs doped with HClO_4_ and VOx-PEDOT as the transparent electrode and hole transport layer, respectively, high-efficiency flexible OSCs were realized with a record high PCE of 8.6%, outstanding mechanical durability, and a high lifetime stability. The new digital printing technology DMD used to deposit PEDOT-SWNT (10:1) doped electrodes and VOx-PEDOT (3:1) as a hole transport layer proposed in this work offers excellent potential for various flexible and highly efficient next-generation optoelectronic devices.
