# Author Correction: Digital printing of a novel electrode for stable flexible organic solar cells with a power conversion efficiency of 8.5%

**DOI:** 10.1038/s41598-021-04283-8

**Published:** 2021-12-27

**Authors:** S. Wageh, Mahfoudh Raïssi, Thomas Berthelot, Matthieu Laurent, Didier Rousseau, Abdullah M. Abusorrah, Omar A. Al-Hartomy, Ahmed A. Al-Ghamdi

**Affiliations:** 1 Department of Physics, Faculty of Science, K. A. CARE Energy Research and Innovation Center, King Abdulaziz University, Jeddah, Saudi Arabia; 2 KELENN Technology, 6 rue Ampère, Igny, France; 3 Electrical and Computer Engineering Department, College of Engineering, K. A. CARE Energy Research and Innovation Center, King Abdulaziz University, Jeddah, Saudi Arabia; 4 Physics and Engineering Mathematics Department, Faculty of Electronic Engineering, Menoufia University, Menouf, 32952 Egypt

Correction to: *Scientific Reports* 10.1038/s41598-021-93365-8, published online 09 July 2021

The original version of this Article contained errors in Figure 8(a) and (b) where the AFM and SEM images were incorrectly constructed showing the electrode on glass, rather than the electrode deposited on PET substrate.

The original Figure 8 and accompanying legend appear below.

**Figure 8:**
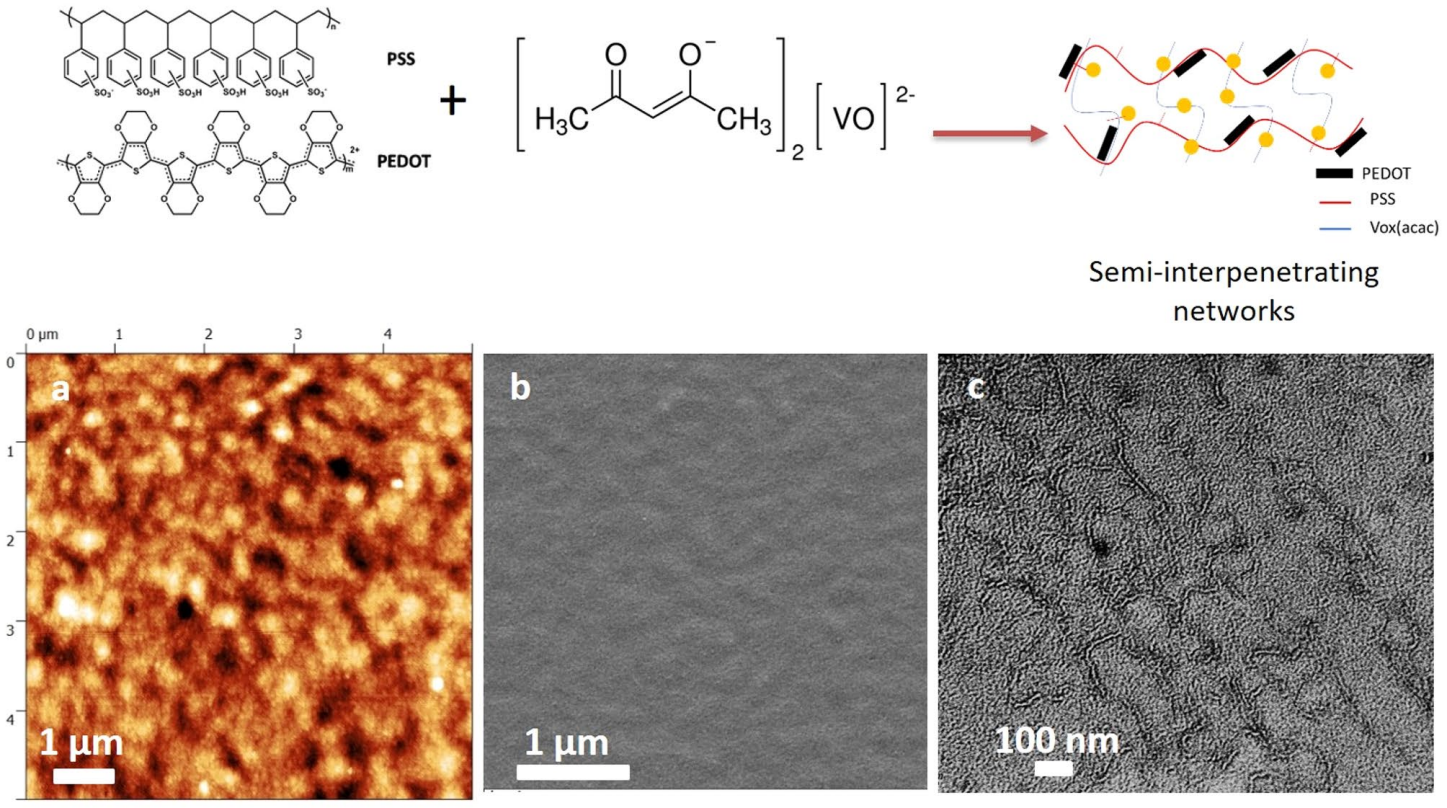
Schematic illustration of the formation of the interpenetrating networks by mixing PEDOT-PSS with VOx(acac). AFM (**a**), SEM (**b**) and TEM (**c**) images of the PEDOT-PSS:VOx (1:3) printed film.

The original Article has been corrected.

